# How Patients Can Contribute to the Assessments of Health Technologies

**DOI:** 10.3390/jmahp13040061

**Published:** 2025-12-15

**Authors:** François Houÿez, Julien Delaye

**Affiliations:** European Organisation for Rare Diseases (Eurordis), 75014 Paris, France; julien.delaye@eurordis.org

**Keywords:** health technology assessment, patent engagement/involvement, community advisory boards, scientific advice, horizon scanning, appraisal, capacity building, mentoring programme

## Abstract

In the process of determining whether a health technology should be covered by healthcare systems, patients and their representatives were initially excluded from both evaluations and decision-making. In Europe, direct dialogue between patient organisations and regulatory authorities—particularly in the pharmaceutical sector—began in the early 1990s. It was only decades later, as the high cost of medicines created new challenges, that authorities recognised the necessity of engaging with patients. Patients’ contributions to the assessment of a health technology begin with discussions about the need for the technology in question. Initially, these discussions involve the developer, and later—after research and development—regulators, HTA assessors, and payers. Given that multiple technologies may be under development, patients and their organisations often prioritise those that generate the most interest within the patient community. They can then share their perspectives with evaluators during the horizon-scanning phase. Another key contribution is the role patients play in guiding clinical research by participating in scientific advice. Finally, during the assessment and appraisal stages, various methods are used to gather their views.

## 1. Introduction

The decision whether to reimburse a health technology was rarely debated publicly in the 20th century in countries with universal health coverage. Debates on high-priced medicines began with biological products for haemophilia, immunoglobulins, and HIV treatments. These discussions arose due to factors such as the high per-patient cost, the overall budget impact, patent-related access challenges in third countries, and the lack of foresight by health authorities regarding pharmaceutical budgets.

Patient and consumer organisations can directly influence medicine prices and take action to reduce costs when they consider prices to be excessive [1]. Interestingly, payers generally do not involve these organisations in price negotiations.

The decision-making process for reimbursement consists of four key steps: the assessment of clinical and social aspects, the evaluation of economic factors, the appraisal, and price negotiation. This article explains how patients can contribute to the first three of these aspects.

## 2. Patients Defining Their Unmet Needs

Asking patients whether their medical needs are met by existing treatments is not a question they can easily answer. Whether severely ill or not, patients expect their disease to be cured. If this is not the case, an unmet medical need is automatically defined. The relative importance of one unmet need compared to another is not their primary concern; patients first consider their own condition in relation to the health status they wish to achieve—what has been described as “achieving or maximising their health potential” [2].

Nevertheless, one of the most significant contributions regulators and health technology assessment (HTA) experts expect from patients is to provide insight into the extent to which a new health technology is needed. Patients, or their representatives, are uniquely positioned to highlight which medical needs are poorly addressed by existing treatments and to identify the limitations of available options. The perceived need for a new medicine, as expressed by patients or their advocates, is an important factor that can influence decision-making.

### 2.1. The Case of People Living with HIV and Growth Hormone

In 2003, the European Medicines Agency (EMA) Scientific Committee for Medicinal Products was evaluating a marketing authorisation application for a growth hormone intended to treat HIV-related cachexia. Although the clinical benefit was modest and the primary endpoint in clinical trials was not met (insufficient weight gain), the product had been designated as an orphan drug. This designation was due to the low prevalence of HIV-related cachexia following the introduction of potent HIV treatments.

One of the EMA committee rapporteurs consulted EURORDIS to gather the opinions and expectations of people living with HIV/AIDS. After consulting with AIDS organisations across Europe, EURORDIS conveyed the following feedback to the committee: “*AIDS groups were not convinced of the utility of recombinant human growth hormone (rhGH) for treating HIV cachexia or AIDS wasting syndrome, even though AIDS wasting remained a reality. One group supported its use, one group opposed it, and the vast majority abstained*” (response to the rapporteur, Eurordis letter to AFSSAPS (French Agency for the Safety of Health Products, name of the French regulatory agency by that time), 8 August 2003).

This input was significant, as AIDS organisations are typically well informed about clinical trial results and the potential effects of products in research and development for HIV infection and opportunistic diseases. Based on this feedback—and despite a few hundred patients having benefited from compassionate use—the committee ultimately decided not to recommend marketing authorisation for this indication. The lack of expressed need from patients and their organisations was a key factor in this decision.

### 2.2. The Case of Children Living with Prader–Willi Syndrome and Growth Hormone

Growth hormones are authorised for various indications, including hormone replacement therapy and the correction of short stature in children with specific genetic conditions (such as Turner syndrome or Prader–Willi syndrome), chronic kidney disease, or those born small for gestational age [3].

Prader–Willi syndrome is a genetic disorder characterised by dysfunctional appetite regulation, leading to an obsession with food and excessive weight gain. Recombinant human growth hormone affects protein, fat, and carbohydrate metabolism, which can help reduce weight by a few kilograms—up to 10 kg in some cases. Initially, this treatment was reimbursed in some EU Member States. However, health technology assessment (HTA) bodies were not universally convinced that reimbursement should continue, given its modest effect on weight in obese children.

In France, the Haute Autorité de Santé (HAS) sought input from the Prader–Willi patient organisation. Patient testimonies highlighted the transformative impact of growth hormone therapy on appetite control. Children treated with the hormone were less obsessed with hunger, which improved their ability to learn and focus. This challenged the outdated characterisation of Prader–Willi syndrome as a form of “*mental retardation*” (as described in a 1956 textbook) [4]. Instead, it demonstrated that the syndrome’s hallmark—an overwhelming preoccupation with hunger—could severely impair learning and attention. With treatment, children showed marked improvements in educational attainment, with some even achieving university degrees. Additionally, the therapy enhanced muscular strength and overall quality of life.

These testimonies helped the HAS recognise the critical need for this treatment in Prader–Willi syndrome and its profound impact on patients’ lives. Consequently, the HAS decided to continue recommending its reimbursement [5].

### 2.3. The Case of End-Stage Heart Disease, an Implantable Heart Device, and Transhumanism

During an early dialogue on the development of an implantable device, patient representatives—potential users of the technology—raised fundamental questions: Was the device truly necessary? Should a new technology be developed simply because it is technologically feasible, or should its necessity be established first? While discussions such as scientific advice or equivalent consultations typically occur at a relatively late stage of development, they often fail to address the core question: Is there a genuine need for the technology?

In Europe, the proposal to revise pharmaceutical legislation includes measures to prioritise “high unmet medical needs”. The aim is to identify areas where public funds could be invested to address these needs. However, beyond the methodological challenges of defining such needs, this approach remains insufficient. There must be clear a priori buy-in from stakeholders. Relying on the market to determine a posteriori whether a technology is useful is not a satisfactory solution. Developing technologies that the market ultimately deems unnecessary incurs costs that could otherwise be allocated to genuinely useful innovations.

This debate intersects with the broader discourse on transhumanism, where medicine moves beyond the traditional realm of treating diseases and enters the domain of augmenting human capacities. Transhumanism is a movement that, leveraging advances in biology and artificial intelligence, advocates for the transformation or surpassing of human limitations to create a post-human or transhuman being with superior capabilities [6].

This raises profound ethical and societal questions: What does humanity gain from implantable heart devices? Is there a limit to how far we should extend life? At what cost—both financial and societal—are we willing to gain a few additional months of life, particularly when such technologies may only be accessible to a privileged few?

### 2.4. Highly Organised Networks Needed

Both HIV/AIDS and Prader–Willi patient groups share a high level of organisation, which enables them to collect data from their members and develop well-founded opinions independently of key opinion leaders or the pharmaceutical industry.

The European Community Advisory Board (ECAB), established by the European AIDS Treatment Group (EATG) in 1997, brings together around 20 patient advocates. These advocates meet regularly with researchers and developers of new treatments to influence R&D programmes and pricing policies—behind closed doors and under confidentiality agreements [7].

Similarly, the French organisation for people living with Prader–Willi syndrome, founded in 1996, quickly became a central hub for affected families in France. By 2003, it had already connected with 251 children, 166 adults, and 176 other individuals associated with the condition.

Patient organisations are often better positioned than individual patients to engage with health technology assessment (HTA) bodies. They can define unmet medical needs and identify the limitations of available treatments by drawing on the collective experiences of their members, or by comparing treatment effects, by listening to patient preferences, and by formulating evidence-based conclusions to share with authorities.

In the context of rare diseases, unmet medical needs can take different forms:

1. Underserved or Neglected Diseases Some rare diseases receive limited research attention, with no products in development. It is estimated that only around 5% of rare diseases have an FDA-approved drug and up to 15% of rare diseases have at least one drug that shows promise in treatment, diagnosis, or prevention [8]. This means that for the vast majority of rare diseases, no specific product has reached Phase II/III clinical trials.
2. Diseases with Limited Treatment Options Even when treatments exist, their effectiveness may be partial or insufficient as not all patients respond to available therapies, treatments may improve some symptoms but not others and side effects or poor tolerability may limit their use. Consequently, many patients still require more effective or safer treatments.

To address these challenges, unmet medical needs should be discussed collaboratively among patients, clinicians, regulators, and HTA bodies. This dialogue ensures that research programmes and investors focus on the most pressing needs for each disease.

A high level of organisation does not require large membership numbers. Even smaller patient organisations can effectively inform decision-makers at critical moments. Training programmes for patient advocates provide both knowledge (“the Know”) and practical skills (“the How”), ensuring meaningful patient involvement in decision-making processes [9].

## 3. Horizon Scanning: Which Products Are Priority?

Patients and their organisations actively engage with researchers from the earliest stages of drug development, and sometimes even during fundamental research. Their involvement can take multiple forms, including:

- Funding research projects directly,
- Organising interdisciplinary scientific discussions with researchers,
- Reviewing the literature to identify research projects aligned with their interests,
- Attending scientific conferences to stay informed about advancements,
- Subscribing to bulletins from learned societies and professional networks.

Organisations with their own Community Advisory Board (CAB) can take this engagement further by inviting academic or industry teams developing medicines or medical devices—from Phase I or even earlier. The CAB provides guidance on all aspects of programme development, ensuring that patient perspectives are integrated from the outset.

### Patient Groups Can Identify Breakthrough Products up to Five Years Before Their Authorisation

Patient groups are often well informed about products in research and development (R&D) long before their marketing authorisation. For example, in the field of HIV, the European Community Advisory Board (ECAB) monitored products of interest an average of five years before their authorisation [10]. Out of 130 products in the HIV pipeline, 18 were selected for in-depth discussions with developers. Of these:

- 9 were authorised,
- 8 were discontinued during the R&D phase, and
- 1 was opposed by advocates, who successfully recommended against its authorisation at an FDA public hearing.

Notably, all HIV products that reached the market during this period had been tracked by the ECAB.

While HIV patient groups were fully aware of which products were nearing authorisation, health authorities were caught off guard in 1996 when highly potent antiretroviral therapies suddenly became available. Having failed to anticipate their arrival, authorities were unprepared to negotiate prices and faced urgent decisions to procure these life-saving treatments. The period from 1995 to the first half of 1996 marked the highest HIV-related mortality in Europe, making rapid access to these therapies critical.

At the time, the European AIDS Treatment Group (EATG), a patient organisation, was the only source of comparative pricing information for HIV medicines across EU Member States.

By alerting health technology assessment (HTA) bodies to emerging technologies years before formal scientific advice or regulatory discussions, patient groups play a unique and invaluable role. They contribute to horizon scanning activities and enable the early detection of innovative treatments, ensuring that health systems are better prepared for their arrival.

## 4. Scientific Advice

As defined by the SEED project (Shaping European Early Dialogues for Health Technology Assessment), the primary objectives of HTA scientific advice or scientific consultations are to minimise the risk that developers submit inadequate or incomplete information to HTA bodies during the assessment of a technology [11].

### 4.1. One Best Practice: Community Advisory Boards (CABs)

Patients can directly contribute their perspectives on the development of health technologies to developers. One of the most effective mechanisms for this is through Community Advisory Boards (CABs). A CAB typically consists of 10 to 20 committed patients and/or carers, appointed by their respective organisations. Unlike industry-led advisory boards, all organisational aspects of a CAB are driven by its members—not by developers. CABs provide independent advice to both public and private sector researchers across a wide range of topics, including:

- Development strategy: Should clinical trials focus on recently diagnosed patients or those with late-stage disease?
- Trial design: Selection of comparators, endpoints, and practical trial organisation.
- Trial conduct: Input on substantial amendments, communication of unexpected side effects, trial termination, and dissemination of results.
- Compassionate use programmes: Organisation and access policies.
- Pricing and reimbursement strategies.

CABs emerged in the 1990s as a response to industry-led advisory boards, where pharmaceutical companies would invite a select few patient advocates to join their committees. The creation of CABs aimed to ensure independence from industry influence and to unify the voices of different patient groups representing the same disease.

A landmark example is TRT-5, established in 1992 in France. This group brought together patient advocates from eight HIV/AIDS organisations (Actions Traitements, Act Up Paris, Aides, Arcat-Sida, Nova Donna, Vaincre Le Sida, Sida Info Services, and Sol En Si). TRT-5 signed an agreement with the ANRS (French National AIDS Research Agency), allowing its members to review all clinical trial protocols for HIV and opportunistic diseases. Simultaneously, the group engaged in dialogues with developers of HIV medicines. The European Community Advisory Board (ECAB), created by the European AIDS Treatment Group (EATG) in 1998, served as the European counterpart to TRT-5.

Published examples demonstrate the impact of CAB feedback on critical aspects of trial design, such as:

- Exclusion criteria, which define the trial population (with potential implications for the P in PICO—Population, Intervention, Comparator, Outcome).
- Use of secondary endpoints, which can influence the O in PICO [12].

CAB discussions are highly relevant for HTA, as they provide a platform for developers and patients to explore unmet needs in the context of existing treatment options—or the limitations thereof. For example:

- The Cystic Fibrosis (CF) CAB, established in 2016, initially focused on the R&D of CFTR modulators (cystic fibrosis transmembrane conductance regulator modulators). These drugs have since transformed the course of the disease for many patients [13]. However, the prevalence of genetic profiles compatible with these treatments varies globally. Future research must now prioritise patients with different genetic mutations who do not benefit from current therapies.
- HIV CAB priorities have evolved over time:
  ○ In the 1990s, the focus was on developing highly active antiretrovirals (HAART) to combat the HIV epidemic.
  ○ Today, priorities include long-acting injectable treatments (administered every six months) to improve lifelong adherence.

Many members of the European HIV/AIDS CAB (EATG) also contribute to European and national guidelines on HIV clinical management. Their insights on new antiretroviral development and treatment strategy trials are invaluable for early HTA dialogues and scientific consultations.

Recent development strategies often involve adding an investigational product to the standard of care and comparing it with the standard alone. However, since the standard of care varies by country, careful consideration must be given to:

- Selection of participating countries to ensure representative subgroup analyses.
- Adequate participant numbers to account for diverse treatment backgrounds.

### 4.2. What Engaging with Patients Entails

Engaging with patients introduces new constraints that differ from collaborations with other experts, such as regulators or HTA professionals. These challenges require adaptability, patience, and a willingness to embrace the distinct dynamics that patient involvement brings. Engaging with patients means:

1. Accepting New Constraints

- Accepting the time it takes: Discussions with patients—even well-trained advocates—require more time than scientific advice provided by regulators or HTA experts. For example, a first meeting with a developer may last an entire day to ensure patient advocates fully understand both the product and the clinical trial design.
- Embracing unpredictable discussions: Unlike structured scientific consultations, patient discussions can take unexpected directions. Patients may raise issues beyond the developer’s prepared questions, reflecting their broader concerns and lived experiences.
- Acknowledging diverse backgrounds: Patients in the room may not always have a scientific background, which necessitates clear communication and patience to ensure mutual understanding.
- Recognising their broader networks: Patients often consult with other stakeholders (e.g., carers, advocacy groups), which can introduce additional perspectives and complexities into discussions.

Other barriers and obstacles to patient engagement exist [14], but there are also recommendations to address them [15].

2. Preparing for Patient-Centred Dialogues

- Co-constructed agendas: Agendas for meetings are prepared collaboratively between developers/sponsors and patient groups (e.g., Community Advisory Boards or CABs). This ensures that patient priorities are included alongside the developer’s questions.
- Two-way dialogue: Unlike traditional scientific advice, patient engagement is a bidirectional process. Patients are free to raise any topic relevant to them, even if it diverges from the developer’s original agenda. For example:
  ○ If a developer proposes a technology for a small patient niche, patients may initiate discussions on pricing policies—even if this topic was not originally planned.
  ○ Questions may extend beyond scientific details to include ethical, practical, or access-related concerns.

3. Practical Considerations for Effective Engagement

To successfully involve patients in HTA scientific advice or early dialogues, the following factors must be considered:

- Language barriers: Finding patient advocates who can understand and express themselves in English (or the working language) can be challenging.
- Preparation time: Adequate time is needed to prepare advocates—ensuring they are informed about the product, trial design, and broader context.
- Inclusivity: Methods of engagement should accommodate diverse levels of expertise and ensure that all voices are heard.

4 Key Steps for Patient Engagement

**Figure 1:**
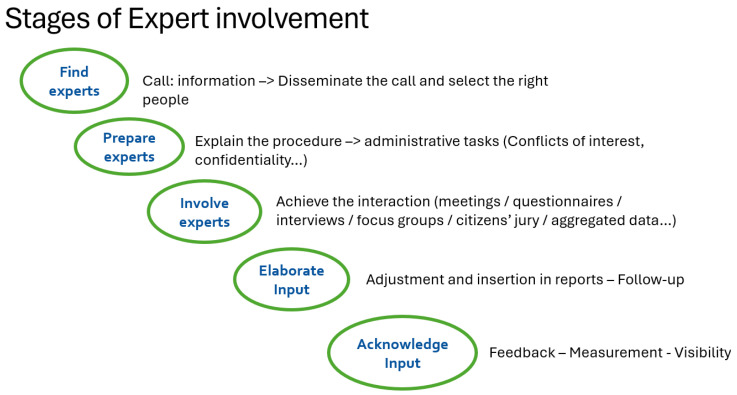
Stages of patient involvement.

The process for involving patients in discussions with developers, scientific advice, or evaluations with regulators/HTA bodies is summarised in Figure 1. These steps apply to all types of procedures, including:

- Developer discussions (e.g., trial design, pricing).
- Scientific advice (e.g., regulatory or HTA consultations).
- Scientific evaluations (e.g., assessments of clinical or economic evidence).

While patient engagement presents challenges, it also offers unique benefits:

- Rich, real-world insights that scientific experts alone may overlook.
- Greater alignment between developed technologies and patient needs.
- Enhanced trust and transparency in the development process.

By embracing these constraints and adapting engagement methods, developers, regulators, and HTA bodies can foster meaningful, productive collaborations with patients.

Engaging patients effectively requires a systematic and respectful process, from identifying the right individuals to ensuring their contributions are meaningfully integrated. Here is a step-by-step breakdown of how to achieve this:

The first resource for finding patients is the membership list of patient organisations. This can be supplemented by databases of individuals known to the organisation, even if they are not formal members. For example, EURORDIS’ database includes contact details for over 4200 individuals, 1095 patient groups across 75 countries (791 in the EU), 84 European or international disease-specific federations, and 54 national alliances.

These resources provide a broad and diverse pool of potential contributors.

Once potential participants are identified, it is essential to discuss their ability to engage effectively in the procedure. While living with a disease provides invaluable firsthand experience, contributing to scientific discussions requires additional skills, such as understanding complex scientific debates, articulating perspectives clearly among experts, navigating technical or regulatory discussions.

Not all patients will have these skills, so this step ensures that those who participate can meaningfully contribute.

Before involvement, patients must be fully informed about the purpose and scope of the procedure they are joining, who is organising it (e.g., developers, regulators, HTA bodies),what constitutes a fruitful contribution from their perspective, documents they will receive and when (e.g., trial protocols, background materials), administrative requirements, such as written declarations of interest, confidentiality agreements, and insider trading prevention measures.

This step ensures transparency and preparedness, helping patients feel confident and informed.

Patients can be engaged through various methods, depending on the context and objectives: phone or virtual interviews for individual input, participation in meetings (e.g., advisory boards, scientific consultations), surveys or questionnaires to gather broader perspectives, focus groups for in-depth discussions, patient juries to evaluate specific questions or dilemmas.

Each method offers unique advantages and can be tailored to the needs of the project.

Once patients have contributed, their input must be elaborated and documented in relevant reports or decision-making materials, reviewed by the patients themselves to ensure accuracy and to allow for any corrections.

This step guarantees that patient perspectives are faithfully represented.

The final steps are critical for maintaining trust and improving future engagements: acknowledgement (recognise patient contributions, either privately (e.g., personalised thank-you notes) or publicly (e.g., in reports or presentations)), satisfaction measurement: (after each interaction, assess the satisfaction of all parties involved—not just patients, but also organisers, developers, and other stakeholders. Evaluate whether mutual expectations were met and identify areas for improvement).

A structured approach to patient engagement ensures that:

- Contributions are meaningful and well-informed,
- Patients feel valued and respected,
- Decisions reflect real-world patient needs and priorities.

By following these steps, organisations can foster productive, mutually beneficial collaborations that enhance the development and evaluation of health technologies.

## 5. Assessment and Appraisal

### 5.1. Ensuring Transparency, Building Trust: Patients Witnessing HTA

Involving patients, their representatives, and civil society in the assessment and appraisal of health technologies serves a fundamental purpose: to ensure transparency, accountability, and trust in decision-making processes. Their participation is indispensable as patients and their representatives act as independent observers, providing a firsthand account of how decisions are made. This role is crucial for building trust: by witnessing the process, patients can explain decisions to their communities, fostering understanding and confidence in the system.

Ensuring evidence-based decisions: patients help verify that decisions are grounded in scientific and medical evidence, rather than industrial, political, or economic influences. This is especially important when patients are denied access to medicines due to insufficient evidence—patients can confirm whether the decision aligns with the scientific rationale or other considerations.

Decisions about reimbursement or pricing are often made at the government level, rather than by HTA bodies alone. Governments may prioritise subsidies for local pharmaceutical companies, effectively providing indirect financial support to national producers. Coverage of therapies with limited evidence (e.g., spa therapy) to support industries that generate significant employment, even if these therapies do not meet evidence-based medicine standards.

Patients serve as a check against such influences, ensuring that public health interests—rather than industrial or political agendas—drive decisions.

Patients can assess the impartiality of other experts involved in HTA procedures. For example, clinicians participating in HTA processes are typically excluded if they were investigators in clinical trials of the technology in question, due to conflict-of-interest policies. However, not being selected as an investigator does not guarantee impartiality. Some clinicians may harbour resentment for not being chosen, which could bias their opinions.

Patients have reported cases where clinicians on appraisal committees opposed a product in discussions yet prescribed it widely under compassionate use programmes. Such observations highlight potential conflicts or inconsistencies that might otherwise go unnoticed.

By participating in assessment and appraisal, patients hold decision-makers accountable for their choices, expose potential biases or conflicts that could undermine the integrity of the process, ensure that patient needs and perspectives remain central to discussions.

Patient involvement is not just about providing input—it is about safeguarding the integrity of health technology decisions. Their presence helps prevent undue influence from industry or political interests, promote fairness and equity in access to medicines, and strengthen public trust in health systems by demonstrating that decisions are transparent, evidence-based, and patient-centred.

### 5.2. Patients as Active Contributors of an HTA and Data They Could Submit

Patients and their representatives play critical and multifaceted roles in the assessment process of health technologies. Their involvement ensures that evaluations are patient-centred, comprehensive, and reflective of real-world needs. They can contribute by defining the Scope of the Assessment: one of the primary roles for patients and their representatives is to help define the questions and scope of the assessment. This includes identifying key issues that matter most to patients, such as unmet needs, treatment priorities, and quality-of-life considerations. It can also include ensuring the assessment addresses relevant outcomes, such as symptom relief, functional improvements, or side effects that significantly impact daily life. Lastly, it can clarify the context in which the technology will be used, including patient preferences and practical challenges.

By shaping the scope, patients help ensure that the assessment focuses on what truly matters to those who will use the technology.

### 5.3. Relevant Patient Outcomes: Relevant, from Whose Perspective?

For the choice of endpoints, patients and their representatives bring unique and indispensable perspectives to the evaluation of health technologies, particularly in defining endpoints, interventions, and quality-of-life measures. Their involvement ensures that assessments are grounded in real-world relevance and reflect what truly matters to those who will use the technology.

Patients should not be limited to commenting solely on quality of life. They are the only ones qualified to determine what is relevant to their own experiences. This includes surrogate markers, which—when truly validated—can significantly impact the efficiency and feasibility of clinical trials.

When HIV RNA was introduced as a surrogate endpoint in 1996, the European AIDS Treatment Group (EATG) presented data to the EMA Scientific Committee, demonstrating that HIV RNA levels could serve as a valid surrogate endpoint for mortality and new AIDS cases. Their evidence showed that each half-log reduction in HIV RNA at 2 months correlated with a 50% reduction in mortality at 1 year, a finding consistent across all antiretroviral products.

The result was the EMA approved this change in 1997, reducing the duration of Phase III clinical trials from 3 to 4 years to just 6 months. The benefits for HTA is that using HIV viral load as a surrogate marker provides equally robust data without loss of information, demonstrating how patient-driven insights can transform assessment processes.

This example highlights that patient-relevant endpoints can extend far beyond quality of life, encompassing scientific and clinical measures that directly impact trial design and regulatory decisions.

While quality of life (QoL) is a critical component of HTA assessments, the tools used to measure it often fall short: generic scales (e.g., EQ5D) may fail to capture disease-specific impacts. Disease-specific scales, even when validated, can miss fundamental effects of treatments that matter to patients.

For Lumacaftor/Ivacaftor for Cystic Fibrosis in 2016, an HTA report noted that the impact on quality of life was only partially documented, focusing solely on the “respiratory symptoms” sub-score of the CFQ-R scale [16]. However, women with cystic fibrosis who received the treatment experienced restored fertility, leading to hundreds of successful pregnancies—an outcome previously unprecedented in the course of the disease. Without timely patient involvement, such life-changing benefits might have been overlooked in clinical development and HTA evaluations.

This underscores the need to involve patients from the scoping phase and in early dialogues, when there is still time to propose additional data collection that reflects patient priorities.

Patients can provide critical insights into the “Intervention” component of the PICO framework, often suggesting alternatives to the developer’s proposed approach. Regarding dosage and formulation, patients may advocate for different doses, formulations, or administration strategies based on their experiences. When an oral formulation was proposed to replace an injectable form (which required hospital visits every 21 days), patients recognised the convenience of the oral option. However, they also raised concerns about potential lower bioavailability and reduced efficacy. Their proposal? Use the oral formulation as a “bridge” between injections, particularly when hospital appointments were difficult to reschedule. This patient-driven strategy ensured maximised efficacy while accommodating practical challenges.

Patient Preference Studies have gained traction among HTA bodies and patient organisations, offering a structured way to quantify what matters most to patients [17]. The Innovative Medicines Initiative (IMI) PREFER project, for example, developed a qualified method for eliciting patient preferences, recognised by the European Medicines Agency (EMA) [18]. The key contributions of Patient Preference Studies are to provide information on:

- Minimum Acceptable Benefit: Patients can specify the minimum treatment effect (e.g., improvement in progression-free survival) required to offset adverse reactions or treatment constraints.
- Risk-Benefit Trade-offs: Studies can reveal how patients weigh risks against benefits, such as accepting certain side effects for improved efficacy or quality of life.
- Comparative Assessments: Patients can compare new treatments against placebos or existing therapies, providing insights into which outcomes they prioritise (e.g., survival vs. quality of life vs. convenience).

Best practices for conducting PPE studies include independent academic leadership, collaboration with patient organisations, timely integration (developers should be encouraged to commission such studies early, so results are available during marketing authorisation evaluations or HTA processes). Some barriers need to be overcome, in particular the time and resources required and the limited number of qualified academic teams capable of designing and conducting these studies.

While patients are not expected to grade the quality of evidence or become semi-statisticians, they play a vital role in interpreting trial results from a patient-centred perspective and can comment on the patient disposition. Patients can provide insights into drop-out rates and the real reasons behind withdrawals (e.g., side effects, logistical challenges).

They can also help contextualising the understanding: Patients can highlight issues reported by trial participants that may not be apparent in the data, such as practical barriers to adherence or unexpected benefits/burdens.

Patient involvement in defining endpoints and shaping assessments ensures that trials and evaluations focus on what truly matters to patients, surrogate markers and quality-of-life measures are relevant and validated, interventions are optimised for real-world use, and decisions are informed by robust, patient-centred evidence.

By actively engaging patients, developers, regulators, and HTA bodies can avoid overlooking critical outcomes and design assessments that reflect the full spectrum of patient needs.

### 5.4. Appraisal: Listening to Different Perspectives

Appraisal is a distinct exercise. Opponents of patient involvement in appraisals often assume that patients will invariably demand reimbursement and that patient organisations are inherently biassed towards advocating for reimbursement regardless of cost. In reality, as seen in marketing authorisation evaluations—where patients frequently express critical views about a product under regulatory review, or at least do not systematically support its authorisation—the same principle applies to the appraisal of technologies for reimbursement decisions.

At the Scottish Medicines Consortium, for instance, patients are invited to comment on the claimed benefits of a new medicine, as presented by the developer, and to assess whether these benefits justify the proposed price. Patients are often the first to criticise a price they consider disproportionate in relation to the expected benefits.

One precondition for involving patients or their organisations in the appraisal process is agreement on the methodology. Organisations that refuse to adhere to the established procedure may be excluded. For example, a patient organisation that rejects the cost-utility approach for economic evaluations (based on cost per QALY) would likely not be invited to participate in appraisals in countries where health technology assessment (HTA) bodies rely on this method, even partially. It would be interesting to explore whether organisations that consider this approach theoretically invalid—as demonstrated by the ECHOUTCOME project—can still participate in the appraisal process [19].

Other principles that could guide patient engagement in appraisal include the following:

- Legitimacy requires that, once accepted, patients and/or consumers be involved with equal credibility to other experts and participants. They should receive the same information and have the same opportunities to express their views.
- Publicity demands that health technology assessment (HTA) procedures—and their conclusions—be clearly understandable, accessible, and verifiable for the broadest possible audience.
- Relevance ensures that the information underlying the assessment must be sufficient to justify the conclusion reached.
- Appeal and revisability necessitate a mechanism that allows for the possibility of an appeal, whether based on new evidence or procedural concerns raised by any party.
- Responsibility entails adhering to the established rules. When patients and/or consumers are consulted appropriately, they should accept the final conclusion, even if it does not align with their preferences.

The latter principle can be particularly challenging to implement. However, once patients and their organisations agree to participate in a procedure, accept its methodology, and are given adequate time to express their views and contribute data, they should respect the outcome—even if dissatisfied. Appraisals are collective processes, shaped by diverse stakeholder perspectives and grounded in the evidence presented in the HTA report. If an effective appeal procedure is in place and the outcome still differs from patient expectations, resorting to media or political channels in an attempt to reverse the decision is considered poor practice.

An important question arises: should the same patients hold a permanent seat on appraisal committees, offering opinions on all procedures—including those involving technologies or conditions outside their expertise? Or should different patients be appointed to appraise technologies relevant to their own conditions?

The workload of appraisal committees presents a significant obstacle. For example, in 2020, the Transparency Committee at the Haute Autorité de Santé held 30 meetings, issued 491 opinions, and adopted 191 summary opinions—an average of 23 opinions per meeting. Given the typical duration of these meetings, each assessment report was discussed for only 16 to 20 min.

## 6. Capacity Building for Patients in HTA for Meaningful Involvement

The importance and relevance of patient engagement in health technology assessment (HTA) have gained increasing recognition in recent years. The new European HTA Regulation (EU) 2021/2282 (HTAR) [20] has emphasised the role of patients in assessing new technologies. According to this Regulation, HTA assessors should consult with patient experts on the scope of the assessment, that is the questions the developer should respond to, based on the PICO approach (Target Patient Population Intervention, Comparators and Outcomes). A second contribution is foreseen when reviewing the European Joint Clinical Assessment Report before it is published. The subgroup conducting the Joint Clinical Assessment has the option of consulting with patients’ organisations, in addition to patient experts. During the development phase, patient experts are expected to take part in Joint Scientific Consultations. Lastly, a Stakeholder Network has been created by the European Commission, with a large number of patient organisations, healthcare professionals, life-science companies…

The European Commission has also highlighted the need for capacity building for patients involved in joint work under the EU4HEALTH programme [21], administered by the European Health and Digital Executive Agency (HaDEA). This programme has partly funded two initiatives aimed at training patients in HTA to prepare them for participation at the European level: the European Capacity Building for Patients (EUCAPA) [22] and the HTA4Patients projects [23].

However, capacity-building opportunities in HTA predate the HTAR in Europe. Examples include EURORDIS—Rare Diseases Europe’s Open Academy programme [24] and initiatives by EUPATI. At the national level, patient organisations in countries such as the Czech Republic, Germany, and France also provide patients with toolkits, support, and educational materials. Similar activities exist in Canada [25], Latin America, Australia [26], and elsewhere. Additionally, some HTA agencies—such as the Haute Autorité de Santé in France and NICE in the UK—offer training materials for patients.

While these training opportunities remain limited overall, there is broad consensus that capacity-building initiatives are essential. They ensure that patients participating in HTA possess sufficient knowledge and skills to engage both actively and meaningfully in the process, whether at the national or European level.

## 7. Conclusions

Involving patients in health technology assessment (HTA) is, above all, a political necessity. Opening the process to civil society is an essential component of responsible governance. By directly observing how technologies are assessed, patients gain a clearer understanding of the process, making them more likely to accept the assessment’s outcome and actively contribute during the appraisal. Witnessing how assessors evaluate data, grade evidence quality, and incorporate input from external experts—including patients and their organisations—helps shift the process away from secrecy and towards greater transparency in decision-making. It is unthinkable, for instance, that the Intergovernmental Panel on Climate Change (IPCC) would operate without the involvement of civil society at every level.

Patient advocates participate in activities at the European Medicines Agency (EMA) on approximately 1000 occasions each year [27], and their contributions are formally recognised. In the interest of full transparency, the EMA publishes the curricula vitae and declarations of interest of patient representatives on its website, as it does for all other experts [28].

Patients and their representatives are, of course, welcome to contribute to HTA discussions, whether during scientific advice sessions, early dialogues, assessments, or other activities. One or two patient experts can provide firsthand accounts of how a new treatment affects their lives or describe the broader impact—both positive and negative—on a larger patient group (through Patient Experience Data or Patient Preferences Elicitation studies). There are often discrepancies between clinical endpoint measurements and patients’ own assessments of benefits and risks.

The precise methods for involving patients in HTA remain experimental. Approaches vary, from face-to-face or telephone interviews and focus groups to participation in meetings, citizens’ or patients’ juries, or written and oral submissions via questionnaires.

A fundamental principle must always apply: patient experts should be involved on an equal footing with other participants. If face-to-face meetings are organised, patients (and clinicians) should have the opportunity to attend in person. Remote written consultations alone prevent patients from fully witnessing the procedure, and the quality of their contribution is optimised only when they can follow the debates from the outset.

A robust mentoring programme is essential. For many patient advocates and organisations, participation in an HTA procedure may occur only once every decade. Even with comprehensive training, most patients involved in a given procedure will likely have had no prior training and only a vague understanding of HTA and its processes. Mentoring can help patients navigate the procedure, review the documents they receive, complete administrative requirements (such as declarations of interest, confidentiality agreements, and insider trading prevention measures), clarify what contributions are expected, and provide support when speaking in public—an experience that can be intimidating.

As identified by Jakab et al., twelve important recommendations for improving the engagement of patients in HTA are consensus [15]. The consensus focused on the involvement in Central and Eastern European Member States but are in fact universal.

To summarise, recommendations include the education of HTA and/or payer organisations on the value and good practices of patient involvement, the acknowledgement of patients as experts on their condition similar to healthcare professionals, the need to revise local HTA guidelines and procedures, the nomination of a dedicated person/team to be responsible for patient involvement activities with sufficient available capacities at each relevant HTA and decision-making body.

Organising meaningful patient engagement ultimately depends on political will and requires investment in both human and logistical resources. Unequivocal institutional commitment is crucial to undertaking this complex but vital activity.

## Data Availability

No new data were created or analyzed in this study.

## References

[1] Houÿez F. (2020). High Price Medicines and Health Budgets: The Role Patients’ and Consumers’ Organisations Can Play. Eur. J. Health Law.

[2] Nord E., Daniels N., Kamlet M. (2009). QALYs: Some Challenges. Value Health.

[3] https://www.ema.europa.eu/en/medicines/human/referrals/somatropin.

[4] Prader A., Labhart A., Willi H. (1956). Ein syndrom von adipositas, kieinwuchs, kryptorchismus und oligophrenic nach myato-mieartigem zustand in neugeborenalte. Schweiz. Med. Wochenschr.

[5] Deal C.L., Tony M., Höybye C., Allen D.B., Tauber M., Christiansen J.S. (2013). The 2011 Growth Hormone in Prader-Willi Syndrome Clinical Care Guidelines Workshop Participants. Growth Hormone Research Society workshop summary: Consensus guidelines for recombinant human growth hormone therapy in Prader-Willi syndrome. J. Clin. Endocrinol. Metab..

[6] Le Bot F., Dard O., Didry C., Dupuy C., Perrin C. (2018). L’homme-machine 2. Du travailleur augmenté à l’homme augmenté. L’Homme et la Société 2018/2, n° 207.

[7] Twenty years of treatment activism. https://www.eatg.org/wp-content/uploads/2021/04/eatg-20-years-report.pdf.

[8] Fermaglich L.J., Miller K.L. (2023). A comprehensive study of the rare diseases and conditions targeted by orphan drug designations and approvals over the forty years of the Orphan Drug Act. Orphanet J. Rare Dis..

[9] Eurordis Open Academy. https://openacademy.eurordis.org/.

[10] Bedlington N., Geissler J., Houyez F., Lightbourne A., Maskens D., Strammiello V., Facey K., Ploug Hansen H., Single A. (2017). Role of Patient Organisations. Patient Involvement in Health Technology Assessment.

[11] Harousseau J., Pavlovic M., Mouas H., Meyer F. (2015). Shaping European Early Dialogues: The Seed Project. Value Health.

[12] Roennow A., Sauvé M., Welling J., Riggs R.J., Kennedy A.T., Galetti I., Brown E., Leite C., Gonzalez A., Portales Guiraud A.P. (2020). Collaboration between patient organisations and a clinical research sponsor in a rare disease condition: Learnings from a community advisory board and best practice for future collaborations. BMJ Open.

[13] Balfour-Lynn I.M., King J.A. (2022). CFTR modulator therapies—Effect on life expectancy in people with cystic fibrosis. Paediatr. Respir. Rev..

[14] Dimitrova M., Jakab I., Mitkova Z., Kamusheva M., Tachkov K., Nemeth B., Zemplenyi A., Dawoud D., Delnoij D.M.J., Houÿez F. (2022). Potential Barriers of Patient Involvement in Health Technology Assessment in Central and Eastern European Countries. Front. Public Health.

[15] Jakab I., Dimitrova M., Houÿez F., Bereczky T., Fövényes M., Maravic Z., Belina I., Andriciuc C., Tóth K., Piniazhko O. (2023). Recommendations for patient involvement in health technology assessment in Central and Eastern European countries. Front. Public Health.

[16] https://www.has-sante.fr/upload/docs/evamed/CT-14927_ORKAMBI_PIC_INS_Avis3_CT14927.pdf.

[17] Germeni E., Fifer S., Hiligsmann M., Stein B., Tonkinson M., Joshi M., Hanna A., Liden B., Marshall D.A. (2024). A genuine need or nice to have? Understanding HTA representatives’ perspectives on the use of patient preference data. Int. J. Technol. Assess Health Care.

[18] Janssens R., Barbier L., Muller M., Cleemput I., Stoeckert I., Whichello C., Levitan B., Hammad T.A., Girvalaki C., Ventura J.J. (2023). How can patient preferences be used and communicated in the regulatory evaluation of medicinal products? Findings and recommendations from IMI PREFER and call to action. Front. Pharmacol..

[19] Beresniak A., Medina-Lara A., Auray J.P., De Wever A., Praet J.C., Tarricone R., Torbica A., Dupont D., Lamure M., Duru G. (2015). Validation of the underlying assumptions of the quality-adjusted life-years outcome: Results from the ECHOUTCOME European project. Pharmacoeconomics.

[20] (2021). Regulation (EU) 2021/2282 of the European Parliament and of the Council of 15 December 2021 on health technology assessment and amending Directive 2011/24/EU. Off. J. Eur. Union.

[21] EU4Health Programme (EU4H) (2022). Call for Proposals Under the Annual Work Programme 2022.

[22] EUCAPA Website. https://www.eucapa.eu.

[23] EUPATI HTA4Patients. https://eupati.eu/hta4patients/.

[24] EURORDIS EURORDIS Summer School. https://openacademy.eurordis.org/summer-school/.

[25] Canada’s Drug Agency (CDA) Website. https://www.cadth.ca/patient-and-community-engagement.

[26] Australian Government Department of Health and Aged Care (2024). Enhance HTA: An Enhanced Consumer Engagement Process in Australian Health Technology Assessment—A Report of Recommendations. https://www.health.gov.au/resources/publications/enhance-hta-an-enhanced-consumer-engagement-process-in-australian-health-technology-assessment-a-report-of-recommendations.

[27] EMA’s Biennial Report on Stakeholder Engagement Activities 2022–2023. https://www.ema.europa.eu/en/documents/report/stakeholder-engagement-report-2022-2023_en.pdf.

[28] European Experts The European Medicines Agency (EMA) Maintains a Public List of European Experts Who Provide Scientific Expertise to EMA’s Activities. https://www.ema.europa.eu/en/about-us/how-we-work/european-medicines-regulatory-network/european-experts.

